# Genome Mining and Characterization of Biosynthetic Gene Clusters in Two Cave Strains of *Paenibacillus* sp.

**DOI:** 10.3389/fmicb.2020.612483

**Published:** 2021-01-11

**Authors:** Jolanta Lebedeva, Gabriele Jukneviciute, Rimvydė Čepaitė, Vida Vickackaite, Raminta Pranckutė, Nomeda Kuisiene

**Affiliations:** ^1^Department of Microbiology and Biotechnology, Institute of Biosciences, Life Sciences Center, Vilnius University, Vilnius, Lithuania; ^2^Department of Analytical and Environmental Chemistry, Institute of Chemistry, Faculty of Chemistry and Geosciences, Vilnius University, Vilnius, Lithuania

**Keywords:** *Paenibacillus*, polyketide synthase, non-ribosomal peptide synthetase, bacteriocin, transcription analysis, RT-qPCR, Krubera-Voronja Cave

## Abstract

The genome sequencing and mining of microorganisms from unexplored and extreme environments has become important in the process of identifying novel biosynthetic pathways. In the present study, the biosynthetic potential of *Paenibacillus* sp. strains 23TSA30-6 and 28ISP30-2 was investigated. Both strains were isolated from the deep oligotrophic Krubera-Voronja Cave and were found to be highly active against both Gram-positive and Gram-negative bacteria. Genome mining revealed a high number of biosynthetic gene clusters in the cave strains: 21 for strain 23TSA30-6 and 19 for strain 28ISP30-2. Single clusters encoding the biosynthesis of phosphonate, terpene, and siderophore, as well as a single *trans*-AT polyketide synthase/non-ribosomal peptide synthetase, were identified in both genomes. The most numerous clusters were assigned to the biosynthetic pathways of non-ribosomal peptides and ribosomally synthesized and post-translationally modified peptides. Although four non-ribosomal peptide synthetase gene clusters were predicted to be involved in the biosynthesis of known compounds (fusaricidin, polymyxin B, colistin A, and tridecaptin) of the genus *Paenibacillus*, discrepancies in the structural organization of the clusters, as well as in the substrate specificity of some adenylation domains, were detected between the reference pathways and the clusters in our study. Among the clusters involved in the biosynthesis of ribosomally synthesized peptides, only one was predicted to be involved in the biosynthesis of a known compound: paenicidin B. Most biosynthetic gene clusters in the genomes of the cave strains showed a low similarity with the reference pathways and were predicted to represent novel biosynthetic pathways. In addition, the cave strains differed in their potential to encode the biosynthesis of a few unique, previously unknown compounds (class II lanthipeptides and three non-ribosomal peptides). The phenotypic characterization of proteinaceous and volatile compounds produced by strains 23TSA30-6 and 28ISP30-2 was also performed, and the results were compared with those of genome mining.

## Introduction

Microorganisms are known to produce a wide range of bioactive compounds that can be used as small-molecule pharmaceutics. These compounds have been reported to exert antibacterial, antifungal, antiviral, antiprotozoal, and anticancer properties. Traditional screening approaches have been used for decades to identify novel bioactive compounds. However, over time, they have become unproductive due to an increasing rediscovery rate (Baltz, 2019; Chen et al., 2019; Männle et al., 2020). Advances in sequencing technologies have allowed us to overcome this problem. As a result, genome sequencing and mining have become very important in the process of identifying novel biosynthetic pathways; tens of thousands of biosynthetic gene clusters (BGCs) have been identified in microbial genomes, most of which encode unknown compounds (Tracanna et al., 2017). In addition, the search for novel bioactive compounds in unexplored and extreme environments has gained traction, and has recently led to the discovery of novel metabolites (Ghosh et al., 2017).

Caves are consistent with the definition of an underexplored and extreme environment. It is estimated that only ∼10% of all caves worldwide have been discovered (Ghosh et al., 2017). The bioactivity of the cave *Actinobacteria* is of special interest since ∼45% of all known microbial bioactive compounds are produced by *Actinobacteria*, ∼80% of which are derived from *Streptomyces* (Ghosh et al., 2017; Belknap et al., 2020). Other taxa, such as *Cyanobacteria*, *Proteobacteria*, and *Firmicutes*, are also known to produce bioactive metabolites, some of which have been successfully commercialized (Ghosh et al., 2017).

The phylum *Firmicutes* belongs to the core cave microbiome and is ubiquitous in caves (Hershey and Barton, 2018). Endospore-forming representatives of this phylum are highly resistant to desiccation and nutrient stress; therefore, they can survive under the harsh conditions of caves (Ikner et al., 2007; Yasir, 2018). For example, in a previous study, we showed that the phylum *Firmicutes* was among the most abundant taxa in Krubera-Voronja Cave, the endospore-forming genera of which (*Aeribacillus*, *Bacillus*, *Caldalkalibacillus*, and *Paenibacillus*) were among the most abundant genera in this deep oligotrophic cave (Kieraite-Aleksandrova et al., 2015). Although the antimicrobial potential of a few endospore-forming cave strains has been characterized (Tomova et al., 2013; Klusaite et al., 2016; Bukelskis et al., 2019), to the best of our knowledge, no study has previously investigated the characterization of the bioactivity of these bacteria at the genome-level.

To fill this knowledge gap, we aimed to perform genome mining for the BGCs of two endospore-forming cave strains from the genus *Paenibacillus*. This genus is known for its potential to produce a wide range of structurally diverse bioactive compounds, including polyketides, peptide-polyketide hybrids, non-ribosomally synthesized peptides, ribosomally synthesized and post-translationally modified peptides (RiPPs) (Cochrane and Vederas, 2016; Grady et al., 2016; Olishevska et al., 2019). The biosynthesis of novel bioactive compounds has yet to be fully elucidated (Zhu et al., 2016a; Park et al., 2017) or predicted through genomic analysis (Aleti et al., 2015; van Belkum et al., 2015; Jeong et al., 2019) in this versatile genus. *Paenibacillus* strains produce a wide range of volatile organic compounds (VOCs) that significantly contribute to the high bioactive potential of these bacteria (Lee et al., 2012; Park et al., 2013; Raza et al., 2015; Cheng et al., 2017; Rybakova et al., 2017; Jeong et al., 2019). It was previously shown that the different strains of *Paenibacillus* differ in the number of BGCs in their genomes as well as in the variety of the bioactive compounds and volatiles produced (Aleti et al., 2015; Jeong et al., 2019). Thus, evaluating the new strains of *Paenibacillus* could lead to the discovery of novel bioactive compounds. In the present study, we aimed to evaluate the biosynthetic potential of two *Paenibacillus* sp. strains using genome mining and the phenotypic characterization of antibacterial activity.

## Materials and Methods

### Isolation, Genotyping, and Identification of Bacterial Strains

Bacterial strains were isolated in our laboratory from sediment samples collected in Krubera-Voronja Cave (43.4184 N 40.3083 E, Western Caucasus) (Kieraite-Aleksandrova et al., 2015). Strains 23TSA30-6 and 28ISP30-2 were isolated as described by Klusaite et al. (2016) at 30°C using tryptic soy agar (TSA) (Merck Millipore, Darmstadt, Germany) and Difco^TM^ ISP medium 4, respectively. Genomic DNA was extracted from fresh grown cultures using the GeneJET Genomic DNA Purification Kit (Thermo Fisher Scientific, Waltham, MA, United States). Genotyping experiments were carried out to exclude the possibility that these two strains represented a single re-isolated strain. BOX-PCR genotyping was performed according to Klusaite et al. (2016). (GTG)_5_-PCR was performed in 50 μL of reaction mixture containing DreamTaq Green PCR Master Mix (2×) (Thermo Fisher Scientific), 0.5 μM (GTG)_5_ primer, and 10 ng of bacterial genomic DNA in an Eppendorf Mastercycler EP Gradient (Eppendorf, Hamburg, Germany). (GTG)_5_-PCR conditions were as follows: initial denaturation at 95°C for 2 min followed by 29 cycles, each consisting of 95°C for 1 min, 53°C for 2 min, and 72°C for 3 min with a final extension step at 72°C for 7 min. BOX-PCR and (GTG)_5_-PCR genotyping profiles were analyzed by electrophoresis on a 1% agarose gel. The amplification of 16S rRNA genes was performed according to Kuisiene et al. (2002). The cloning of the 16S rRNA genes and sequence analysis were performed as described by Klusaite et al. (2016). The 16S rRNA gene phylogenetic tree was constructed using the MEGA7 program (Kumar et al., 2016) with the maximum likelihood method. The size of the 16S rRNA gene used for alignment was 1368 nt. Bootstrap analysis of the maximum likelihood data using 1000 re-samplings was carried out to evaluate the validity and reliability of the tree topology. 16S rRNA gene sequences were deposited in GenBank under accession nos. MK511842 and MK511843. The whole genome sequences (*see below*) of both strains were used for digital DNA–DNA hybridization (dDDH). A genome-to-genome distance calculator^1^ (GGDC 2.1; Meier-Kolthoff et al., 2013) was used for dDDH. The results of formula 2 were adopted according to a previous study (Meier-Kolthoff et al., 2013). Strains 23TSA30-6 and 28ISP30-2 were deposited in the German Collection of Microorganisms and Cell Cultures GmbH (DSM 109041 and DSM 109042, respectively).

### Evaluation of Antimicrobial Activity of Bacterial Strains

The primary screening of the antibacterial activity of strains 23TSA30-6 and 28ISP30-2 was performed using a cross-streak assay, as described by Klusaite et al. (2016). Four test strains representing both Gram-positive and Gram-negative bacteria were used for screening: *Micrococcus luteus* Sar1 (*Actinobacteria*), *Bacillus thuringiensis* TL8 (*Firmicutes*), *Escherichia coli* BL21(DE3) (*Proteobacteria*), and *Pseudomonas* sp. VR1 (*Proteobacteria*).

An agar-well diffusion assay was used to characterize the proteinaceous antimicrobial compounds. TSA plates with cut wells were prepared as described by Klusaite et al. (2016). The test strain *M. luteus* Sar1 was used as an indicator for this analysis. Cells from the liquid culture were removed by centrifugation (7000 × *g*, 20 min, 4°C). Serial two-fold dilutions of the supernatant were prepared using 25 mM Tris-HCl buffer (pH 7). A volume of 100 μL of each dilution was poured into the prepared wells and TSA plates were incubated at 35°C for 24 h. The antimicrobial activity was expressed in arbitrary units (AU) as the maximum dilution that produced a clearly visible zone. The unit of antimicrobial activity (AU) was defined as the reciprocal of the highest level of dilution resulting in a clear zone of growth inhibition (Pranckutė et al., 2015).

### Cultivation of Bacterial Strains and Extraction of Antibacterial Compounds

1× and 0.5× tryptic soy broth (TSB) (Merck Millipore) was used to cultivate strains 23TSA30-6 and 28ISP30-2. Inoculation (1% vol/vol) was performed using fresh bacterial cultures grown on TSA plates at 30°C. Inocula (OD_590_ = 1.6) were prepared in the respective sterile growth medium. Bacteria were cultivated in an orbital shaker Multitron Standard (Infors AG, Bottmingen-Basel, Switzerland) at 30°C and 180 rpm. The samples were aseptically removed every 2 h to determine culture growth. The growth of the culture was monitored by measuring the optical density at 590 nm. The experiments were repeated eight times. Cells from the liquid culture were removed by centrifugation (7000 × *g*, 20 min, 4°C). The supernatant was used for the extraction of antibacterial compounds.

Volatile and semivolatile compounds in the supernatant were extracted from cultures cultivated in 1 × TSB until the exponential-stationary growth phase transition. Extractions were performed with equal volumes of chloroform, methanol, or hexane in a separating funnel (Jiang et al., 2016). The organic solvent extracts were evaporated to dryness and dissolved in chloroform. These extracts were further subjected to gas chromatography-mass spectrometry (GC-MS) analysis.

Antibacterial proteinaceous compounds were salted-out with ammonium sulfate (80% saturation) from cultures cultivated in 1 × TSB until the exponential-stationary growth phase transition. The precipitate was recovered by centrifugation (20000 × *g*, 50 min, 4°C), then dissolved in 25 mM Tris-HCl buffer (pH 7), dialyzed for 72 h at 4°C against the same buffer using the SnakeSkin Dialysis Tubing, 3.5K MWCO (Thermo Fisher Scientific), and concentrated by polyethylene glycol 35000 (Sigma-Aldrich, St. Louis, MO, United States). The protein concentration was determined using the Pierce Coomassie (Bradford) Protein Assay Kit (Thermo Fisher Scientific).

### GC-MS Analysis of Antibacterial Compounds

The GC analysis of supernatant extracts was performed on a PerkinElmer Clarus 580 series gas chromatograph coupled to a PerkinElmer Clarus 560 S mass spectrometer (PerkinElmer, Shelton, CT, United States). The GC system was equipped with an Elite-5MS capillary column (30 m × 0.25 mm i.d., 0.25 μm film thickness) coated with methylpolysiloxane (5% phenyl). Helium was employed as a carrier gas with a constant flow of 1 mL min^–1^. The GC conditions were as follows: the oven temperature was programmed at 40°C for 1 min, from 40 to 250°C at 3°C min^–1^, and held at 250°C for 8 min; the injector temperature was held at 250°C. Injection was performed in pulsed splitless mode (pulsed to 4 mL min^–1^ until 1.5 min, split (10:1) open at 1.55 min). The capillary column was connected to the ion source of the mass spectrometer by means of a transfer line maintained at 280°C. The electron ionization ion source conditions were as follows: electron energy 70 eV and temperature 180°C. GC-MS in full scan mode was used. The analyses were carried out with a filament multiplier delay of 3 min, and the acquisition was performed in the range of *m/z* 33–600. The qualitative identification of the different compounds was performed by comparing their mass spectra with those stored in the NIST (National Institute of Standards and Technology) library. GC-MS identified compounds were compared with the bacterial VOCs included in the Microbial Volatile Organic Compounds 2.0 database^2^ (Lemfack et al., 2018).

### Characterization of Proteinaceous Antimicrobial Compounds

Tricine-SDS-PAGE (T-SDS-PAGE) was used to fractionate proteinaceous antibacterial compounds. T-SDS-PAGE was performed according to the protocol described by Schägger (2006). Protein (400 μg) was loaded onto wells. After electrophoresis, half of the gel was fixed (50% methanol, 12% acetic acid) for 2 h, and the protein bands were visualized by staining with PageBlue Protein Staining Solution (Thermo Fisher Scientific). The other half of the gel was used for zymogram analysis. It was fixed for 2 h (20% 2-propanol, 10% acetic acid), soaked in deionized water for 2 h, placed in a sterile Petri dish, and covered with TSA containing 10^6^ CFU mL^–1^ of the test strain *M. luteus* Sar1. The plate was incubated at 35°C for 24 h. To determine the molecular mass of proteinaceous antimicrobial compounds, the inhibition zones were aligned with the corresponding bands on the T-SDS-PAGE gel. To identify the compounds, the bands were excised from the gel and subjected to liquid chromatography coupled to tandem mass spectrometry (LC-MS/MS) analysis. LC-MS/MS was performed at the Mass Spectrometry Laboratory of the Institute of Biochemistry and Biophysics, Polish Academy of Sciences.

The thermostability of the proteinaceous compounds was investigated at 40, 50, 60, 70, 80, 90, and 100°C, and the samples were incubated for 30 min. The samples were also autoclaved at 121°C for 15 and 30 min. The pH stability was examined at pH 5–10 after incubation at 4°C for 4 h. The following buffers were used: 100 mM sodium acetate (pH 5–6), 100 mM phosphate (pH 7), and 100 mM Tris-HCl (pH 8–10). The pH of the samples was adjusted to neutral before testing their antimicrobial activity, as described by Pranckutė et al. (2015). The proteinaceous compound samples were treated with various enzymes at a final concentration of 1 mg mL^–1^, including proteinase K, α-chymotrypsin, papain, trypsin (AppliChem, Darmstadt, Germany), α-amylase (Merck Millipore), β-chymotrypsin, ficin, pepsin, pronase E, and lipase (Sigma-Aldrich), dissolved in buffers as recommended by the supplier. During enzymatic treatment, the samples were incubated at 37°C for 2 h. After incubation, the enzymatic reactions were stopped by heating at 95°C for 5 min. In all tests, the remaining antibacterial activity was evaluated using the agar-well diffusion assay and expressed in AU (*see above*).

### Whole Genome Sequencing and Mining of BGCs

Whole genome sequencing and genome sequence assembly were performed at GenXPro GmbH (Frankfurt am Main, Germany). These Whole Genome Shotgun projects were deposited at DDBJ/ENA/GenBank under accession nos. SPQH00000000 (28ISP30-2) and SLTL00000000 (23TSA30-6). The versions described in this paper are versions SPQH01000000 and SLTL01000000. Assembly metrics were computed using QUAST v4.4 (Gurevich et al., 2013). Completeness and contamination of assemblies were estimated using CheckM v. 1.0.18 (Parks et al., 2015). The characteristics of the assembled genomes are shown in Table 1. The genome mining of the BGCs was performed using the antiSMASH 5.0 pipeline^3^ (Blin et al., 2019), BAGEL3, and BAGEL4 web server^4^ (van Heel et al., 2018). BGCs were further analyzed using MEGA7 (Kumar et al., 2016) and the BLAST tool^5^, as well as the MIBiG database (v. 2.0)^6^ (Kautsar et al., 2020), Bactibase database^7^ (Hammami et al., 2010), and the bioinformatics tool NaPDoS^8^ (Ziemert et al., 2012).

**TABLE 1:** The characteristics of the assembled genomes.

Characteristics	SLTL00000000 (23TSA30-6)	SPQH00000000 (28ISP30-2)
Total assembly length (bp)	5,750,436	5,238,062
G+C content (%)	46.8	46.9
Number of contigs	139	3687
Contig N50	168,556	1,968
Sequencing depth of coverage	123.0×	3.5×
Completeness (%)	99.85	81.65
Contamination (%)	0.0	3.36

### RNA Extraction and Synthesis of cDNA

For RNA extraction, the bacterial cultures were cultivated as described above. The strains were all sampled from the middle of the exponential growth phase, at the transition phase, and at the beginning of the stationary growth phase. RNA extraction was performed using the GeneJET RNA Purification Kit (Thermo Fisher Scientific), and the integrity of RNA samples was analyzed by electrophoresis on a 1% agarose gel.

Total RNA samples were treated with dsDNase (Thermo Fisher Scientific) to remove any DNA contamination. To test for leftover DNA contamination in RNA samples, the samples were tested by 16S rRNA gene PCR (*see above*). For reverse transcription PCR, the Maxima First Strand cDNA Synthesis Kit for RT-qPCR (Thermo Fisher Scientific) was used. First-strand cDNA was synthesized using 1 μg of the DNA-free RNA template and random hexamer primers.

### Transcription Analysis

The Primer-BLAST tool^9^ (Ye et al., 2012) was used to construct the quantitative PCR (qPCR) primers targeting the biosynthetic genes identified in the genomes of strains 23TSA30-6 and 28ISP30-2. The size of the expected PCR products was ≤200 bp, and melting temperature of the primers was in the range of 58–60°C. All of the primer pairs were verified by PCR using the genomic DNA of both strains as the template. PCR was carried out in 50 μL of reaction mixture containing DreamTaq Green PCR Master Mix (2×) (Thermo Fisher Scientific), 0.25 μM each primer, and 10 ng of genomic DNA. The PCR conditions were as follows: initial denaturation at 95°C for 3 min followed by 30 cycles, each consisting of 94°C for 30 s, 55°C for 30 s, and 72°C for 30 s, with a final extension step at 72°C for 5 min. The PCR products were purified using the GeneJET PCR Purification Kit (Thermo Fisher Scientific) and sequenced at the DNA Sequencing Center (Vilnius University, Life Sciences Center, Lithuania). The sequences of the PCR products were analyzed using MEGA7 software and the BLAST tool. Only those primer pairs that resulted in high-quality target-specific sequences were used for transcription analysis.

The SensiFAST SYBR No-ROX Kit (Bioline, London, United Kingdom) was used for transcription analysis. The amplifications were performed on a CFX96 Touch^TM^ Real-Time PCR Detection System (Bio-Rad, Berkeley, CA, United States) in a standard two-step protocol, according to the manufacturer’s instructions. qPCR was carried out in 20 μL of reaction mixture containing 10 μL of 2 × SensiFAST SYBR No-ROX Mix, 0.8 μL of each primer (10 μM), and 2 μL of cDNA template under the following conditions: polymerase activation at 95°C for 3 min followed by 40 cycles each consisting of 95°C for 5 s and 60°C for 30 s. The amplification specificity was confirmed by melt curve analysis. The thermal profile for melt curve determination was initiated with incubation at 65°C, followed by a gradual increase in temperature (0.5°C/5 s) up to 95°C. The absolute quantification approach was used in the qPCR experiments. The calibration curves were constructed according to Bukelskis et al. (2019). The amplifications were analyzed using the supporting CFX Manager software (v. 2.1) (Bio-Rad). The results were converted to the number of copies of a template using the calculator to determine the number of copies of a template^10^. Heat maps showing the results of the transcription analysis were constructed using the Heatmapper^11^ web server (Babicki et al., 2016).

## Results

### Identification of Bacterial Strains

The bacterial strains 23TSA30-6 and 28ISP30-2 were isolated from sediment samples collected from Krubera-Voronja Cave. Both strains were highly active against Gram-positive test strains and *E. coli* BL21(DE3) in a cross-streak assay, but they differed in their activity against the test strain *Pseudomonas* sp. VR1: 28ISP30-2 inhibited the growth of the latter strain, while 23TSA30-6 did not.

**FIGURE 1:**
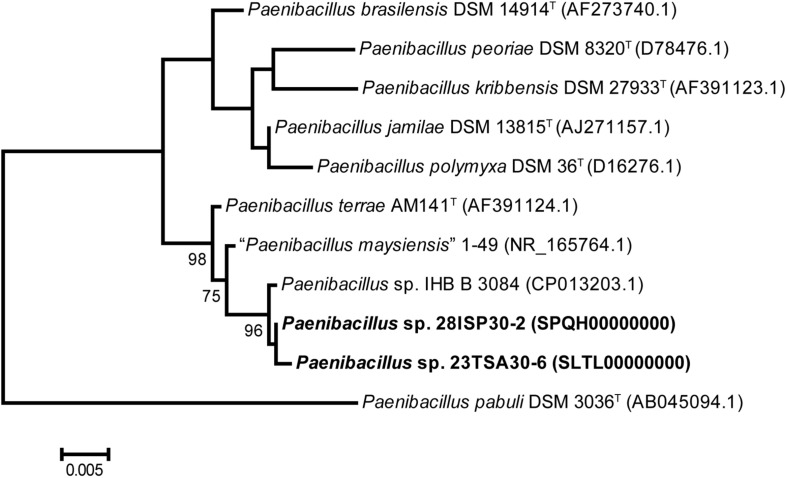
Phylogenetic positions of strains 23TSA30-6 and 28ISP30-2, as well as the nearest neighbors from the genus *Paenibacillus*. The numbers at the nodes represent the percentage of bootstrap values obtained from 1000 samplings. Only the most significant values (>70%) are presented. The tree was rooted using the 16S rRNA gene sequence of *Paenibacillus pabuli* DSM 3036^T^ as an outgroup. Scale bar: 0.005 nucleotide substitution per site.

For the genotyping of these two strains, both BOX-PCR and (GTG)_5_-PCR genotyping techniques were used. The BOX-PCR electrophoretic profiles were identical, while those of (GTG)_5_-PCR differed slightly (data not shown). Consequently, it was concluded that the strains 23TSA30-6 and 28ISP30-2 represent two different strains of the same species. The 16S rRNA gene BLAST search showed that both strains belonged to the genus *Paenibacillus*. The highest sequence similarity of the 16S rRNA gene was with that of *Paenibacillus* sp. IHB B 3084 (99.87% for strain 28ISP30-2 and 99.80% for strain 23TSA30-6) and *Paenibacillus terrae* AM141^T^ (99.07% for strain 28ISP30-2 and 99.13% for strain 23TSA30-6), as well as with that of *Paenibacillus maysiensis* 1–49 (99.03% for strain 28ISP30-2 and 98.89% for strain 23TSA30-6). All these sequences also constituted a separate cluster in the phylogenetic tree (Figure 1). The sequence similarity with the 16S rRNA gene of the other species of *Paenibacillus* was ≤98.22%.

In order to assign the strains 23TSA30-6 and 28ISP30-2 to the species of the genus *Paenibacillus*, dDDH was carried out using genomic sequences. According to Chun et al. (2018), only strains showing ≥98.7% 16S rRNA gene similarity should be selected for dDDH. Our analysis showed that both strains from the Krubera-Voronja Cave belonged to a single species, with a dDDH value of 86.2%. dDDH with the genomic sequence of *Paenibacillus* sp. IHB B 3084 was also >70.0% (84.1% and 81.6% for the strains 23TSA30-6 and 28ISP30-2, respectively). However, this value was <70.0% (63.5% for 23TSA30-6 and 62.4% for 28ISP30-2) when the genome of strain *P. maysiensis* 1–49 was used for analysis. Unfortunately, the genomic sequence is unavailable for the type strain AM141^T^ of the species *P. terrae*. To overcome this problem, dDDH was performed with all the genomes that were attributed to the species *P. terrae* in the databases. The dDDH value >70.0% was obtained in only one case, for the genome of strain *P. terrae* GHS.8NWYW.5; it was 84.5% for strain 23TSA30-6 and 82.1% for strain 28ISP30-2. For the other four genomes of *P. terrae* (accession nos. CP003107.1, JTHP00000000.1, PNXQ00000000.1, and VEHP00000000.1), the dDDH values were in the range of 20.9–59.6% for 23TSA30-6 and 22.7–59.4% for 28ISP30-2. It is interesting to note that the dDDH value was <70.0% between these four genomes of *P. terrae* and the genome of *P. terrae* GHS.8NWYW.5. Therefore, strains 23TSA30-6 and 28ISP30-2 could not be unambiguously assigned to the species *P. terrae.* However, our results clearly showed that four strains represent the same species in the genus *Paenibacillus*, namely *Paenibacillus* sp. 23TSA30-6, *Paenibacillus* sp. 28ISP30-2, *Paenibacillus* sp. IHB B 3084, and *P. terrae* GHS.8NWYW.5.

### Characterization of Volatile and Semivolatile Compounds

Our previous results showed that the antimicrobial activity of the cave strains was dependent on the VOCs emitted by these strains (Klusaite et al., 2016). In the present study, GC-MS analysis was used to evaluate the volatile and semivolatile compounds produced by 23TSA30-6 and 28ISP30-2. The results of this analysis are shown in Supplementary Table 1. In total, 33 different compounds were reliably identified in the extracts of culture supernatants from the cave strains. Among these, 21 compounds were detected in the extracts of both strains. The most common compounds were classified as fatty acid derivatives, while three compounds were classified as nitrogen-containing VOCs (Caulier et al., 2019). Both non-cyclic [octadecanamide and (*Z*)-octadec-9-enamide] and cyclic (3-benzyl-2,3,6,7,8,8a-hexahydropyrrolo[1,2-a] pyrazine-1,4-dione) nitrogen-containing compounds were detected. The latter compound is also known as cyclic dipeptide cyclo(Pro-Phe). Another three compounds [2,6,11-trimethyldodecane, 2,7,10-trimethyldodecane, 2,6,11,15-tetramethylhexadecane (also called crocetane)] from the extracts of both cave strains were classified as members of acyclic terpenes.

In the extracts of strain 23TSA30-6, an additional five unique compounds were identified (Supplementary Table 1). One of them (hexadecanamide) was classified as a non-cyclic nitrogen-containing VOC, while the remaining four compounds of strain 23TSA30-6 were classified as fatty acid derivatives.

An additional seven compounds were detected in the extracts of strain 28ISP30-2 (Supplementary Table 1). Among the fatty acid derivatives, a few isoprenoid alkanes (2,6,10-trimethylpentadecane (also called norpristane), 4,8-dimethylundecane, 2,6,10,14-tetramethylheptadecane, 2,6,10,15-tetramethylheptadecane) were identified. One sulfur-containing VOC [(1-methyl-2,2-diphenylcyclopropyl) sulfanylbenzene] was also detected in the extracts of strain 28ISP30-2, whereas no unique nitrogen-containing VOC was found in them.

### Characterization of Proteinaceous Antibacterial Compounds

**FIGURE 2:**
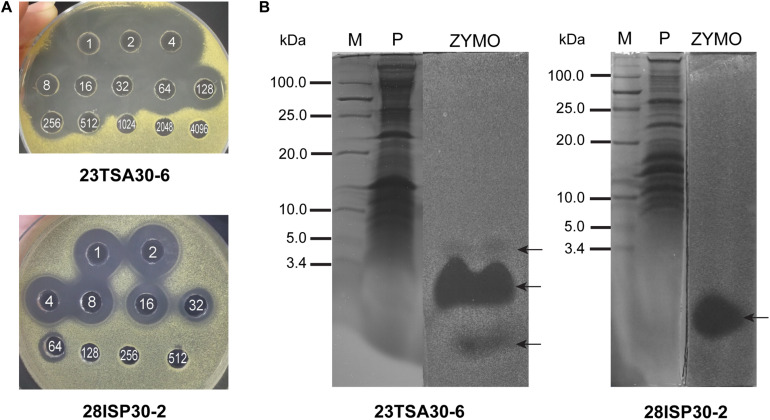
Characterization of proteinaceous antibacterial compounds. **(A)** An agar-well diffusion assay of crude preparations. The white numbers in the wells denote the dilution of the crude preparation. **(B)** Comparison of T-SDS-PAGE gel and zymogram of crude preparations. M, *PageRuler Unstained Low Range Protein Ladder* (Thermo Fisher Scientific); P, T-SDS-PAGE gel; ZYMO, zymogram. The test strain *M. luteus* Sar1 was used as an indicator for this analysis. The zones of antimicrobial activity against the test strain are indicated by black arrows.

To identify other compounds that are likely to contribute to the high antibacterial activity of the cave strains 23TSA30-6 and 28ISP30-2, salting-out of the proteinaceous compounds was carried out. The bioactivity of these compounds was examined using an agar-well diffusion assay. The crude preparation of strain 23TSA30-6 demonstrated the antimicrobial activity of 1383.8 AU mg^–1^ of protein, while the antimicrobial activity of strain 28ISP30-2 was lower (914.3 AU mg^–1^ protein) (Figure 2). This difference in activity of the crude preparations could be caused by the number of the active compounds – zymogram analysis revealed three antimicrobial compounds in the crude preparation of strain 23TSA30-6, while only one was detected for strain 28ISP30-2. The comparison of T-SDS-PAGE and the zymograms showed that one compound of strain 23TSA30-6 was ∼4 kDa in size, while the other two compounds of this strain, as well as the single compound of strain 28ISP30-2, were <3.4 kDa (Figure 2).

Liquid chromatography coupled to tandem mass spectrometry analysis was performed to identify the detected proteinaceous compounds, but failed. Three peptides were predicted in the gel slices corresponding to the two smaller compounds of strain 23TSA30-6, but the BLASTP search did not allow us to relate them to any bioactive compound produced by the genus *Paenibacillus* (data not shown). No peptide was predicted for the antimicrobial compound of strain 28ISP30-2 nor for the largest compound of strain 23TSA30-6.

The crude preparation of the proteinaceous compounds of strain 23TSA30-6 was investigated in more detail. The results of temperature and pH stability assays showed that this preparation retained 100.0% of its activity after incubation at pH 5–10 and at 40–60°C. Incubation at 70–100°C and autoclaving at 121°C for 30 min resulted in 50.0% residual activity. Treatment with proteinase K and pronase E completely eliminated the antibacterial activity of the crude preparation, whereas treatment with other proteolytic enzymes, α-amylase, and lipase had no impact on its activity. These results allowed us to confirm the proteinaceous nature of antimicrobial compounds in the crude preparation and to conclude that these compounds have no carbohydrate or lipid moieties in their structure.

### Characterization of the Identified BGCs

To determine the BGCs responsible for the high antimicrobial activity of strains 23TSA30-6 and 28ISP30-2, the genomes of these strains were sequenced and genome mining was performed. BGCs were detected in 23 contigs of strain 28ISP30-2 and 33 contigs of strain 23TSA30-6. As a whole range of contigs contained only fragments of BGCs, such that we could not exclude the possibility that they represented the different fragments of the same BGC, the contigs were compared to each other in order to determine the exact number of BGCs in the genome of each strain. When BGCs were predicted to encode the biosynthesis of the same type of metabolite [for example, non-ribosomal peptide synthetase (NRPS)], the high sequence similarity of the clusters was the main parameter with which to assign biosynthetic regions to the same BGC. Genome mining of the closely related *Paenibacillus* sp. IHB B 3084 was also performed, and the BGCs identified were compared with those of both strains from the Krubera-Voronja Cave. The genome of *Paenibacillus* sp. IHB B 3084 (Dhar et al., 2016) was chosen because it is a complete genome, compared to the genome of the other phylogenetically related strain *P. terrae* GHS.8NWYW.5 (Marcolefas et al., 2019), which is not; instead, it is at a contig assembly level. The genome mining results are shown in Table 2.

**TABLE 2:** Summary of antiSMASH 5.0- and BAGEL4-based metabolite prediction in *Paenibacillus* sp. strains 23TSA30-6, 28ISP30-2, and IHB B 3084.

Metabolite type	IHB B 3084	23TSA30-6	28ISP30-2	References
Phosphonate	1	1	1	
Siderophore	1	1	1	
Terpene	1	1	1	
*Trans*-AT PKS/NRPS	1	1	1	
NRPS-like-1	1	1	0	
NRPS-like-2	0	0	1	
NRPS (total)	7	10	9	
NRPS-1	1 (plasmid pHD01)	1 (NRPS, betalactone)	1	
NRPS-2	1 (plasmid pHD02)	1	1	
NRPS-3	1 (plasmid pHD03)	1	1	
NRPS-4 (fusaricidin)	1 (chromosome 2475410–2543796)	1	1	Li et al., 2007
NRPS-5 (polymyxin B)	1 (chromosome 3414655–3493942)	1	1	Choi et al., 2009
NRPS-6	1 (chromosome 5195939–5248344)	0	0	
NRPS-7 (tridecaptin)	1 (chromosome 5397000–5490523)	1	1	Lohans et al., 2014
NRPS-8 (colistin A)	0	1	1	Tambadou et al., 2015
NRPS-9	0	1	1	
NRPS-10	0	1	0	
NRPS-11	0	1	1	
Bacteriocin of proteusins family	1	1	1	
Lanthipeptide-1, class I	0	1	1	
Lanthipeptide-2, class I (paenicidin B)	1	1	1	Lohans et al., 2014
Lanthipeptide, class II	0	1	0	
Lasso peptide, class II	1	1	1	
Sactipeptide-1	1 (chromosome 2854535–2874535)	0	0	
Sactipeptide-2	1 (chromosome 3905987–3925987)	1	1	

*The most similar known cluster is shown in parentheses.*

Single BGCs encoding the biosynthesis of phosphonate, terpene, and siderophore were identified in all three genomes. Both the sequence similarity of the core biosynthetic genes and the structure of the clusters were highly similar in the different genomes. A single hybrid *trans*-AT PKS/NRPS was also identified in all analyzed *Paenibacillus* sp. genomes (Table 2). The hits of the MIBiG database search were found to be BGC of paenilipoheptin (Vater et al., 2018), but had a low (52.0–82.0% depending on the module) sequence identity. The NaPDoS-based analysis of the ketosynthase (KS) domain of *trans*-AT PKS/NRPS showed that its sequence similarity with the sequences in the database was ≤59.0%. However, a similarity of >85.0% of the KS domains at the amino acid level is needed to predict the putative products of PKS (Ziemert et al., 2012). The substrate amino acid of the adenylation domain (AD) of the hybrid PKS/NRPS was predicted to be D-glycine. It should be noted that this amino acid was not previously identified in paenilipoheptin (Vater et al., 2018).

**FIGURE 3:**
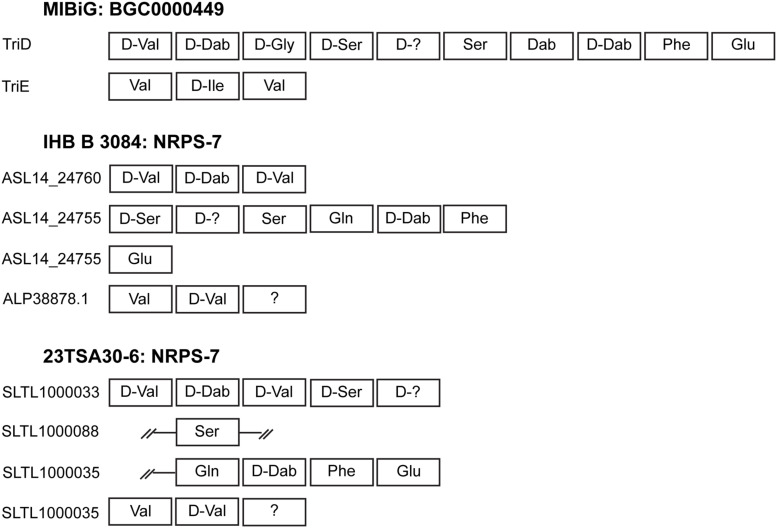
Schematic representation of the modules and ADs identified in NRPS-7. The representative BGC000049 of tridecaptin from the MIBiG database is shown for comparison. Incomplete regions of the modules are indicated by double strokes.

Most of the BGCs identified could be assigned to the NRPS. The cave strains had a larger number of NRPSs than the genome of strain IHB B 3084 (Table 2). NRPS-4, NRPS-5, NRPS-7, and NRPS-8 were predicted to represent known biosynthetic pathways of *Paenibacillus* sp. (Table 2). A comparison with the MIBiG database showed that the core biosynthetic enzyme of NRPS-4 was the most similar to fusaricidin synthetase ABQ96384.2 (Li et al., 2007) of *Paenibacillus polymyxa* (97.0–98.0% of amino acid sequence identity). The modules of NRPS-5 were the most similar to PmxA, PmxB, and PmxE from the polymyxin B biosynthetic pathway (Choi et al., 2009) of *P. polymyxa* (97.0–99.0%, 98.0%, and 97.0–100.0% of amino acid sequence identity, respectively). NRPS-8 showed a high (96.0–98.0% sequence identity depending on the strain) similarity with PmxA, which is involved in the biosynthesis of other polymyxin – polymyxin E_1_, also called colistin A (Tambadou et al., 2015). It should be noted that isoleucine-specific AD was identified in NRPS-8, but not in NRPS-5 (Table 3). This finding, together with the differences in the sequences of NRPS-5 and NRPS-8, clearly indicates that the genomes of cave strains 23TSA30-6 and 28ISP30-2 encoded two different polymyxins. The proteins encoded by the core biosynthetic genes of NRPS-7 were the most similar to TriD (79.0–94.0% of identity depending on the module) and TriE (85.0% identity) from tridecaptin BGC from *P. terrae* (Lohans et al., 2014). However, the structural organization of the modules and ADs in these modules differed significantly in the predicted tridecaptin BGCs (Figure 3). The only identical module was the module with three ADs specific for valine, D-valine, and one unidentified amino acid.

**TABLE 3:** Predicted monomers and products of BGC of *Paenibacillus* sp. strains 23TSA30-6 and 28ISP30-2.

BGC	Predicted monomers and products	Predicted molecular mass (Da)
NRPS-like-1	Phe	
NRPS-like-2	Ser	
NRPS-1	Ile + D-X + Val	
NRPS-2	Orn + Gly + D-X + D-X + Ser + Pro	
NRPS-3	D-X (Phe/Trp) + X (Phe/Trp)	
NRPS-4 (fusaricidin)	Thr + D-X (Ile/Val) + X (alloIle/Ile/Val) + D-Thr + D-Asn + Ala	
NRPS-5 (polymyxin B)	D-X (Phe/Trp) + Thr + Dab + Dab + Thr + Dab + Thr + D-Dab + Dab + Dab	
NRPS-7 (tridecaptin)	D-Val + D-Dab + D-Val + D-Ser + D-X + Ser + Gln + D-Dab + Phe + Glu + Val + D-Val + X	
NRPS-8 (colistin A)	D-X (Phe/Trp) + Ile + ?	
NRPS-9	? + D-X + Val	
NRPS-10	Gln + ?	
NRPS-11	? + Pro	
Lanthipeptide-1, class I	MAKFDDFDLDVVSVSKQGSGFEPQ- VDhbDhaKDhaLCDhbPGCVDhbGILQDhbCALQDhbIDhbCNCHIDhaK MKKFDDFDLDIISVSKEVTHFEPQ- IDhbDhaIDhaLCDhbIGCNDhbDhaFLKCLN MANFDDFDLDIVTASKKNSNFEPR-IEDhaLLFCDhbPGCADhbGVVLCPK	3162.8 2023.4 1997.4
Lanthipeptide-2, class I (paenicidin B)	MADNLFDLDVQVNKSQGSVEPQ- VLDhaIVACDhaDhaGCGDhaGKDhbAADhaCVADhbCGNKCFDhbNVGDhaLC	3237.8
Lanthipeptide, class II	MSKKEMIRALRDPEFRKQFEDFSHPSGVVSEDELKQVVGG -AELDhbPNDhaIIVDhbLGDhbGDhaGCKDhbYYVCDhbKDhbNDCIA VGGFYFSLESSTGG- NNQDhaHFALILLLDhaKDhaWLQRCGCYGILIDGVKNARRLIL	3193.6 4274.1
Lasso peptide, class II^a^	MSKKEWQEPTIEVLDINQTMA-**GKGWKEID**WVSDHDADLVNPS	2350.5

*“?” denotes the end of the incomplete NRPS cluster located on the contig edge when the presence/absence of AD was impossible to determine. Predicted leader peptides of RiPPs are underlined. ^*a*^Putative macrolactam is bolded. Dha, didehydroalanine; Dhb, didehydrobutyrine; X, unknown substrate amino acid of AD.*

**FIGURE 4:**
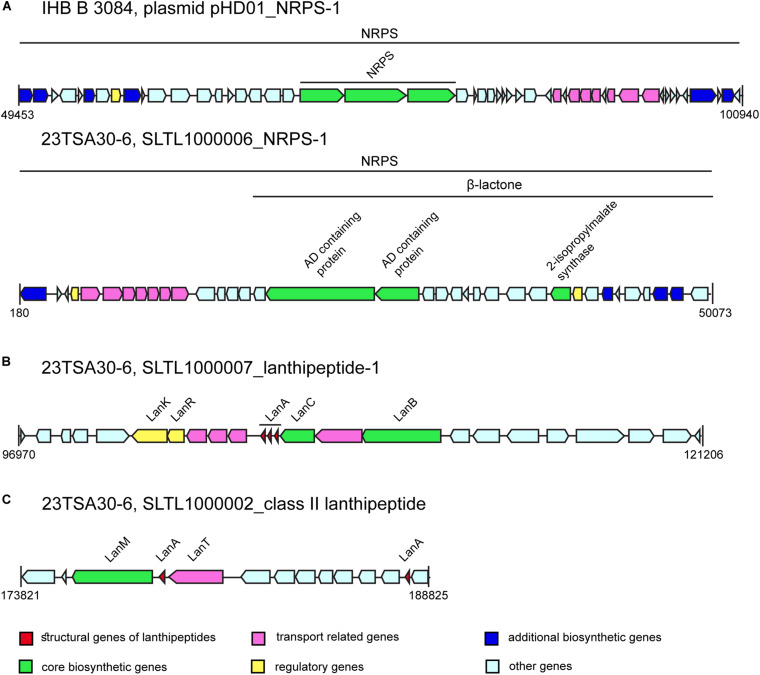
The BGCs of bioactive compounds (not drawn to scale). **(A)** NRPS-1. **(B)** Lanthipeptide-1. **(C)** Class II lanthipeptide.

One of the most interesting BGCs in the *Paenibacillus* sp. genomes investigated was NRPS-1. The MIBiG database search showed a very low sequence identity of the different NRPS-1 modules with proteins from completely different BGCs: PlpE from pelgipeptin BGC (Qian et al., 2012) from *Paenibacillus elgii* (55.0% sequence identity), spumigin E BGC (Fewer et al., 2009) from *Nodularia spumigena* (37.0% identity), paenilipoheptin (Vater et al., 2018) from *P. polymyxa* (33.0% identity), and TriE from tridecaptin BGC (Lohans et al., 2014) from *P. terrae* (32.0% identity). The sequence similarity between NRPS-1 from the analyzed genomes was high (97.9–99.19% depending on the module and domain), although the structural organization of this NRPS in strains IHB B 3084 and 23TSA30-6 differed (Figure 4). Two fragments of NRPS-1 were found on the different contigs of the genome of 28ISP30-2 (data not shown); therefore, we could not determine which structural organization of NRPS-1 – whether that of strain IHB B 3084 or that of strain 23TSA30-6 – was characteristic of NRPS-1 in strain 28ISP30-2. The predicted monomers of the product of this NRPS are shown in Table 3. However, it should be noted that the final product can differ because of an additional core biosynthetic gene located in the BGC of NRPS-1 in strain 23TSA30-6 (Figure 4). This gene encodes 2-isopropylmalate synthase, which is involved in the biosynthesis of β-lactone (Robinson et al., 2019). It was also identified in the upstream region of NRPS-1 in the genome of strain IHB B 3084, but antiSMASH-based analysis did not assign it to the common BGC with NRPS-1 (Figure 4). The gene encoding 2-isopropylmalate synthase was also found upstream of NRPS-1 in the genome of 28ISP30-2. However, due to the short contig on which this gene is located, we could not evaluate its involvement in the biosynthesis of β-lactone.

The remaining NRPSs (NRPS-2, NRPS-3, NRPS-9, NRPS-10, and NRPS-11) could not be assigned to any known BGC. They showed an amino acid sequence identity of 44.0-71.0% with NRPSs involved in the biosynthesis of paenibacterin (Huang et al., 2014), paenilipoheptin (Vater et al., 2018), pelgipeptin (Qian et al., 2012), and tridecaptin (Lohans et al., 2014). Different modules of NRPS-2 demonstrated similarity with the NRPSs from two different pathways involved in the biosynthesis of paenilipoheptin and tridecaptin. The predicted monomers of the putative products produced by these NRPSs are shown in Table 3.

Each examined genome contained a single NRPS-like gene (Table 2). The NRPS-like protein of strain 23TSA30-6 was highly (99.14%) similar to that of strain IHB B 3084; phenylalanine-specific AD was found in these NRPS-like proteins (Table 3). The MIBiG database search showed a very low (31.0%) sequence identity with BogE from bogorol A BGC (Dejong et al., 2016) from *Brevibacillus laterosporus*. The NRPS-like protein of strain 28ISP30-2 differed significantly from that of strain 23TSA30-6, with an amino acid sequence similarity of only 39.06%. The NRPS-like protein of strain 28ISP30-2 was the most similar (84.0% sequence identity) to TriD from tridecaptin BGC (Lohans et al., 2014); serine-specific AD was identified in this NRPS-like protein (Table 3).

RiPPs constituted another group of metabolites for which biosynthetic enzymes were identified in the analyzed genomes of *Paenibacillus* sp. (Table 2). BGCs responsible for the biosynthesis of class I and class II lanthipeptides, class II lasso peptides, sactipeptides, and bacteriocins of the proteusins family were identified.

A single BGC of class II lasso peptide was found in all of the genomes investigated. The amino acid sequence of the predicted RiPP is shown in Table 3. It was identical in both cave strains and showed a sequence similarity of 100.0% with *Paenibacillus* multispecies paeninodin family lasso peptide WP_149094479. Its sequence similarity with paeninodin (Zhu et al., 2016b) itself was only 45.95%. An eight-residue macrolactam ring was predicted in this lasso peptide.

The BGCs of two different class I lanthipeptides were identified in the genomes of the cave strains, while the genome of IHB B 3084 encoded only one lanthipeptide of this class (Table 2). The amino acid sequence of the structural gene of lanthipeptide-2 was found to be the most similar to PabA from paenicidin B BGC (Lohans et al., 2014) from *P. terrae* (98.0% identity). The BGC of lanthipeptide-1 was identified only in the genomes of the cave strains. This BGC encodes three different structural genes of the lanthipeptides (Figure 4 and Table 3). Comparison with the MIBiG database showed that the largest lanthipeptide was the most similar to NisA from nisin A BGC (Trmčić et al., 2011) from *Lactococcus lactis* subsp. *lactis*, but with a very low sequence identity (56.0%). The two smaller lanthipeptides were the most similar to the structural genes from ericin S BGC (Stein et al., 2002) from *Bacillus subtilis* (56.0% and 79.0% of amino acid sequence identity). The presence of post-translationally modified amino acid residues didehydroalanine (Dha) and didehydrobutyrine (Dhb) was predicted for all class I lanthipeptides (Table 3).

The BGC of class II lanthipeptide was found only in the genome of the cave strain 23TSA30-6 (Table 2). Two structural genes of RiPP were identified in this cluster (Figure 4 and Table 3). The database search did not reveal any significant similarity for the larger lanthipeptide. The smaller lanthipeptide showed a very low (53.85%) similarity with *Paenibacillus fonticola* hypothetical protein WP_019638037. Both lanthipeptides were predicted to contain post-translationally modified amino acid residues Dha (both peptides) and Dhb (only the smaller peptide).

The BGC of bacteriocin from the proteusins family was also predicted in all investigated genomes (Table 2), and the precursor of this bacteriocin was found to be identical to that of the NHLP leader peptide family natural product precursor WP_149096110 of *P. terrae*. The biosynthesis of two different sactipeptides was predicted for strain IHB B 3084, while the cave strains appeared to only have the potential to produce one of them (Table 2). The precursor of sactipeptide-2 had no hits in the non-redundant protein database, but the nucleotide sequence of this putative ORF overlapped with *Paenibacillus* multispecies acyl carrier protein WP_014277736.1. It should be noted that this precursor was identified using BAGEL3 as a substrate peptide suitable for modification but was not found using BAGEL4; thus, the false prediction of this precursor could not be completely excluded. The final structures of the sactipeptides and proteusins were not predicted.

### Transcription Analysis of BGCs

The biosynthesis of bioactive compounds always starts from the transcription of the respective genes. In order to determine which BGCs encoded in the genomes of *Paenibacillus* sp. were transcribed, quantitative transcription analysis was performed. For this analysis, the primers targeting the BGCs of the cave strains were designed (Table 4). The only exception was the BGC of siderophore, and the primers targeting this BGC were not constructed. It should be noted that despite our repeated attempts to design specific primers, chimeric or poor-quality sequences were obtained for BGCs encoding NRPS-like-2, NRPS-10, class I lanthipeptide-1, and bacteriocin of the proteusins family. Consequently, the transcription analysis of these BGCs was not carried out.

**TABLE 4:** Primers designed and used for transcription analysis.

Primer pair	Sequence (5′→3′)	Target	PCR product size (bp)	Strain and genome contig used for construction of the primer pair
G1	F: GGCGGTCGGGGAACTATATG	NRPS-1, adenylation domain	169	23TSA30-6; SLTL01000006
	R: TAGCCATTTCGCCCGATCTC			
G2	F: AGACAGGGGCGATTGTGTTC	NRPS-7, adenylation domain	174	23TSA30-6; SLTL01000033
	R: GATTGAACAGTGGCGCAGTC			
G3	F: AGATTGCACCGCTCGTTCAT	*Trans*-AT PKS/NRPS, ketosynthase domain	164	23TSA30-6; SLTL01000034
	R: CACCACATGGCAGTTTGTCC			
G5	F: AGACAGCCGCATCAAGTGAA	NRPS-11, adenylation domain	181	23TSA30-6; SLTL01000006
	R: AGGTTACACAGCCCACGATG			
G7	F: TCGTCCCGATTGGAAAGGTG	NRPS-9, adenylation domain	191	23TSA30-6; SLTL01000062
	R: CAGACGCTCTCCACTCACAA			
G8	F: CCACACTGTACGTACCGACC	NRPS-2, adenylation domain	181	23TSA30-6; SLTL01000065
	R: AGCTCTAATGAGGAGGCCGA			
G12	F: TCCGCTCGTAGCTTTCAAGG	NRPS-5	166	23TSA30-6; SLTL01000064 28ISP30-2; SPQH01000139
	R: ATGAATATCCAGCGCGAGCA			
G20	F: CGCGTGAAAACAGACCCTTG	Paenicidin B-like class I lanthipeptide dehydratase	186	23TSA30-6; SLTL01000015 28ISP30-2; SPQH01000021
	R: GCGTTCTCGACTTGGATGGA			
G21	F: TCTAAATCCGGGCACCGAAG	NRPS-8, adenylation domain	194	23TSA30-6; SLTL01000053 28ISP30-2; SPQH01000454
	R: CGAAAATCGCTCCGTCGAAC			
G22	F: TTGAACGGTCCCCGCATTTA	*Trans*-AT PKS/NRPS, adenylation domain	175	23TSA30-6; SLTL01000077
	R: GCTCGCCTGGTACACCAATA			
G26	F: GCACTGGCTCATGGCTATCT	NRPS-like-1, adenylation domain	172	23TSA30-6; SLTL01000007
	R: TGCCCCGGATTTTGAGTTGT			
G27	F: CTTCTGAGAAACTGGCGGGT	NRPS-4, adenylation domain	162	23TSA30-6; SLTL01000022 28ISP30-2; SPQH01000112
	R: GTAAGCGCTAAGTGTGGGGA			
G28	F: TTGCGATGGTAACCCGACTT	NRPS-3	163	23TSA30-6; SLTL01000006
	R: TTCAAGGGATAACGTCCGGC			
G29	F: GCTTCCTGGACGCAGGTATT	Phosphoenolpyruvate mutase	175	23TSA30-6; SLTL01000005 28ISP30-2; SPQH01000001
	R: TGGGGAACCGCTTATCTTCG			
G30	F: ATCATGGCGGACCGGAATTT	Terpene cyclase	155	23TSA30-6; SLTL01000007
	R: GTTCCGAGGAGTCTGCTGAG			
RB4	F: TGGCTGACAATTTATTTGATCTGGA	Structural gene of paenicidin B-like class I lanthipeptide	167	23TSA30-6; SLTL01000015 28ISP30-2; SPQH01002101
	R: TGAACCGACGTTGGTAAAGCA			
RB6.2	F: CACCCAACGAAAGGCTCATC	Class II lanthipeptide synthetase LanM	200	23TSA30-6; SLTL01000002
	R: TCCCTGAGCCGTATCAAAGTAAA			
RB7	F: TGTCATTCACGGTGAGGAAGT	Lasso peptide biosynthesis, PqqD family chaperone	160	23TSA30-6; SLTL01000001 28ISP30-2; SPQH01000047
	R: CAGAGCAGCTACGATCTCCC			
RB9	F: TCCTCCACTTGCAGCATGAA	Putative structural gene of sactipeptide-2	160	28ISP30-2; SPQH01000727
	R: CACAGGGAGGAGTTGCTATGC			
RB10	F: GGCGCGAAATGACCTTTTGT	Sactipeptide-2 modification, radical SAM protein	192	23TSA30-6; SLTL01000003 28ISP30-2; SPQH01000727
	R: GGTCACGCAGTTCAATCAGC			
RB11	F: TGTGACGGCCCAAAATACGA	Lasso peptide biosynthesis, nucleotidyltransferase family protein	182	23TSA30-6; SLTL01000001 28ISP30-2; SPQH01000047
	R: ACAAGCGGGTCCAATCCAAT			

**FIGURE 5:**
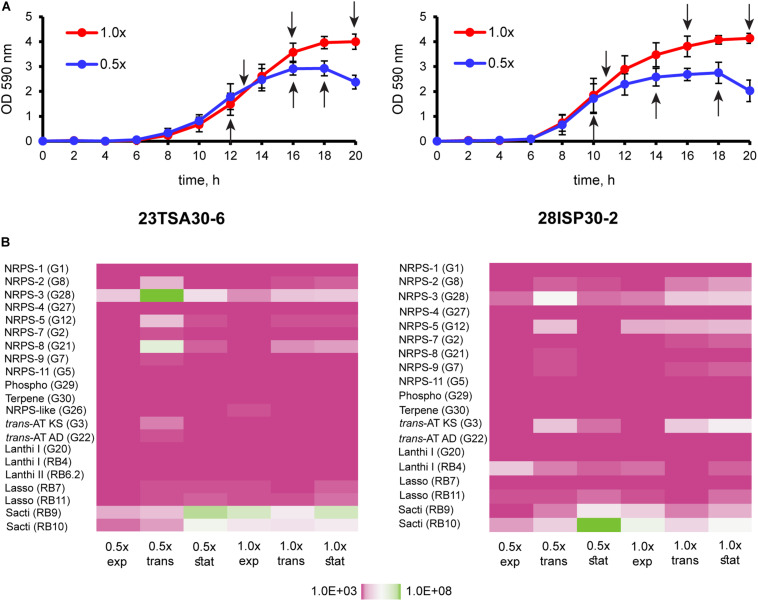
Transcription analysis of BGCs of the cave strains 23TSA30-6 and 28ISP30-2. **(A)** Growth curves of the cave strains 23TSA30-6 and 28ISP30-2. Sampling points for RNA extraction are indicated by black arrows. **(B)** Heat maps showing the results of transcription analysis. The primer pair used in qPCR is indicated in parentheses. 1× and 0.5×, cultivation in 1 × TSB and 0.5 × TSB, respectively; exp, the exponential growth phase; *trans*, the transition phase; stat, the stationary growth phase; phospho, phosphonate; *trans*-AT KS and AD, *trans*-AT PKS/NRPS ketosynthase and adenylation domain, respectively; lanthi I, paenicidin B-like class I lanthipeptide-2; lanthi II, class II lanthipeptide; lasso, lasso peptide; sacti, sactipeptide-2.

For transcription analysis, the cave strains were cultivated in TSB (Figure 5). Two different concentrations of nutrients were used: 1 × TSB and TSB with the reduced amount of nutrients, that is, 0.5 × TSB. As the genomes of the cave strains were predicted to encode both primary and secondary bioactive metabolites, the handling of the samples for transcription analysis was performed at several different culture growth phases: in the middle of the exponential growth phase, at the exponential-stationary growth phase transition, and at the beginning of the stationary growth phase. Transcription analysis showed that all BGCs encoded in the genomes of the cave strains were transcribed under all examined conditions, although the level of transcription differed (Figure 5). The most intensively transcribed BGCs in both strains were those of NRPS-3 and sactipeptide-2. The transcription of NRPS-2, NRPS-5 (polymyxin B), NRPS-8 (colistin A; strain 23TSA30-6), *trans*-AT PKS/NRPS, and paenicidin B-like lanthipeptide-2 (strain 28ISP30-2) was determined to be less intensive.

## Discussion

In the present study, the biosynthetic potential of *Paenibacillus* sp. strains 23TSA30-6 and 28ISP30-2 was investigated. Both strains were isolated from the deep oligotrophic Krubera-Voronja Cave and were highly active against both Gram-positive and Gram-negative bacteria.

Genome mining revealed a high number of BGCs in the cave strains: 21 for strain 23TSA30-6 and 19 for strain 28ISP30-2. These numbers are higher than those previously reported for *Paenibacillus* sp. genomes (Jeong et al., 2019). It was previously determined that strains of the same microbial species usually encode a core set of BGCs, but they can also encode additional clusters that can vary significantly from strain to strain (Baltz, 2019; Belknap et al., 2020). Our results were in agreement with this observation, namely that the genomes of strains 23TSA30-6 and 28ISP30-2 encoded the biosynthesis of the full range of common metabolites. However, the strains also differed in the presence of some NRPSs and the clusters encoding biosynthesis of some RiPPs. It is important to note that only a small portion of the predicted BGCs could be attributed to the biosynthesis of known compounds of *Paenibacillus* sp.

The whole range of NRPSs was predicted in the genomes of 23TSA30-6 and 28ISP30-2. *Paenibacillus* strains capable of producing several different non-ribosomal peptides were also described previously (Selim et al., 2005; Aleti et al., 2015; van Belkum et al., 2015). Some of the predicted NRPSs could be assigned to the biosynthesis of known compounds, including fusaricidins, polymyxins, and tridecaptins. Fusaricidins have been reported to show high activity against Gram-positive bacteria and fungi, but only weak activity against Gram-negative bacteria, while tridecaptins and polymyxins are well-known for their high levels of activity against Gram-negative bacteria (Grady et al., 2016; Olishevska et al., 2019). On the other hand, one of the polymyxins of *Paenibacillus* sp. strain B2 was reported to be active against Gram-positive bacteria, and this activity was hypothesized to be associated with the unusual amino acid composition of this peptide (Selim et al., 2005). The predicted BGCs of tridecaptin and polymyxins in the genomes of the cave strains indicated that these peptides may be responsible for the antibacterial activity of these strains against the test strains of *E. coli* and *Pseudomonas* sp. However, the predicted unusual first amino acid (Phe/Trp instead of L-Dab) (Lohans et al., 2012; Tambadou et al., 2015) in both polymyxins may change the conventional spectrum of activity of these peptides. Alternatively, an unusual first amino acid can also decrease the polymyxin production to undetectable low titers, diminishing or completely eliminating the antibacterial activity of the produced compounds (Winn et al., 2016; Stanišić and Kries, 2019). Leucine instead of L-Dab in the first position was also identified in polymyxin of *P. polymyxa* E681; however, its impact on bioactivity was not determined (Jeong et al., 2019).

The BGC of tridecaptin was unusual in terms of both the structural organization of the modules and the substrate specificity of ADs. To the best of our knowledge, four modules instead of the conventional two modules of tridecaptin BGC were not previously reported (Cochrane et al., 2015; Jeong et al., 2019). D-Val instead of Gly in the third position, as well as Gln instead of Dab in the seventh position, suggests that the genomes of the two cave strains and strain IHB B 3084 encode a new analog of tridecaptin (Lohans et al., 2014; Cochrane et al., 2015; Cochrane and Vederas, 2016; Jeong et al., 2019). The spectrum of bioactivity of this analog remains to be elucidated.

The other predicted NRPSs may produce novel peptides. The most intriguing was the BGC of NRPS-1. The structural organization of this cluster suggested that β-lactone ring formation occurred during the biosynthesis of non-ribosomal peptide. To the best of our knowledge, β-lactone-containing natural products have never been identified in the genus *Paenibacillus*, although they have been reported for some other taxa (Kreitler et al., 2019; Robinson et al., 2019).

Despite the large number of *Paenibacillus* sp. genomes in the databases, the number of characterized ribosomally synthesized peptides in the databases remains small. The MIBiG and BAGEL4 databases only contain information on six of them: paenibacillin, paenicidins A and B, penisin, paeninodin, and paenilan. The Bactibase database contains additional elgicins, bacillocin 602, and bacillocin B37. All of these except both bacillocins belong to class I RiPPs, namely class I lanthipeptides and class II lasso peptides (Teng et al., 2012; Arnison et al., 2013; Acedo et al., 2018). Both bacillocins were previously attributed to class II non-modified peptides (Abriouel et al., 2011). The genome mining of the 23TSA30-6 and 28ISP30-2 strains revealed BGCs of only class I RiPPs. It should be noted that only lanthipeptide-2 could be associated with the known BGC of *Paenibacillus*, namely with the cluster encoding biosynthesis of paenicidin B. Our results showed that the genomes of the strains 23TSA30-6 and 28ISP30-2 encode novel, currently unknown RiPPs with a low sequence similarity with the entries in the databases. Recently, BGCs of class II lanthipeptides were shown to be present in the genomes of *Paenibacillus* sp. for the first time; their occurrence is rare in these genomes (Baindara et al., 2020). A rare structural feature was also determined for the BGCs of lanthipeptide-1 and class II lanthipeptides of the cave strains—multiple structural genes were identified in these BGCs. Similarly, multiple structural genes have previously been detected in BGCs of lanthipeptides in some other *Paenibacillus* sp. genomes (Baindara et al., 2020) as well as in the BGCs of lanthipeptide ericin (Stein et al., 2002), sactipeptide thurincin H (Lee et al., 2009), and some circular bacteriocins (Vezina et al., 2020). This type of structural organization is common only for two-peptide lantibiotics (Abriouel et al., 2011).

The genome mining results correlated with those of the characterization of proteinaceous compounds. These compounds were sensitive to digestion with proteinase K and pronase E, stable over a wide range of pH, relatively thermostable, and active against the Gram-positive test strain *M. luteus* Sar1. These results suggest that RiPPs, particularly lanthipeptides and/or sactipeptides, were successfully produced. Lanthipeptides and sactipeptides are usually sensitive to proteinase K (Rea et al., 2010; Lajis, 2020); all known lanthipeptides of *Paenibacillus* sp. have been reported to be active against Gram-positive bacteria, while penisin is also known to be active against Gram-negative bacteria (He et al., 2007; Lohans et al., 2012; Baindara et al., 2016; Park et al., 2017), similar to the sactipeptides and polytheonamide B of the proteusins family, which have also been previously found to be active against Gram-positive bacteria (Lee et al., 2009; Rea et al., 2010; Freeman et al., 2012; Chiumento et al., 2019). We supposed that lasso peptides were transcribed but were not translated and/or modified, at least in strain 23TSA30-6. Lasso peptides are known to be highly stable against proteolytic digestion and are usually highly thermostable. Therefore, it is likely that they were not present in the crude preparations (Hegemann et al., 2015). On the other hand, we cannot rule out the possibility that the lasso peptide of strain 23TSA30-6 was produced but was not active against the test strain. However, in our opinion, this possibility was unlikely, since the antimicrobial activity against Gram-positive bacteria is common for lasso peptides (Tan et al., 2019).

The molecular mass of the largest proteinaceous compound of strain 23TSA30-6 correlated well with that predicted for the larger class II lanthipeptide. The BGC of this RiPP was not predicted in the genome of strain 28ISP30-2. The other two zones of inhibition of strain 23TSA30-6 correlated well with the two ranges of the predicted molecular masses of lanthipeptides: ∼2 kDa (1997.4–2023.4 kDa) and ∼3.2 kDa (3162.8–3237.8 kDa). The comparison of the genome mining results and zymogram analysis allowed us to suppose that lanthipeptide-1 was successfully produced in strain 23TSA30-6 but was not translated and/or modified in strain 28ISP30-2. The production and activity of this lanthipeptide should result in two zones of activity. However, for unknown reasons, strain 28ISP30-2 only successfully produced lanthipeptide-2 (paenicidin B) but not lanthipeptide-1, whereas strain 23TSA30-6 produced all three predicted lanthipeptides. Unfortunately, the failure of LC-MS/MS analysis did not allow us to confirm this conclusion.

Gas chromatography–mass spectrometry analysis of the volatile and semivolatile compounds was carried out to evaluate the probable impact of these compounds on the antimicrobial activity of strains 23TSA30-6 and 28ISP30-2. Most of the VOCs identified in both strains have been previously reported in antimicrobial mixtures of bacteria (Raza et al., 2015; Sheoran et al., 2015; Mishra et al., 2017) and fungi (Dos Reis et al., 2019) volatiles, as well as in plant extracts with antimicrobial activity (Abukabar and Majinda, 2016). Strain 23TSA30-6 additionally emitted dibutyl benzene-1,2-dicarboxylate, the antibacterial activity of which was demonstrated in a previous study (Roy et al., 2006). Hexadecane was identified in the mixtures of the volatiles of both cave strains; this antibacterial compound was previously reported to be produced by *P. polymyxa* strain E681 (Park et al., 2013). However, the most intriguing characteristic of the volatiles of both strains was the production of acyclic terpenes. It is worth noting that the BGC of terpene biosynthesis was predicted in the genomes of both strains; the transcription of this BGC was also found to occur in both strains. The terpene biosynthetic pathway is unknown in the genus *Paenibacillus* sp. The partial hypothetical pathway was proposed by Passera et al. (2018) for *Paenibacillus pasadenensis* strain R16, which was reported to produce sesquiterpenoid farnesol. The antibacterial activities of sesquiterpenoids and different terpenes against Gram-positive and Gram-negative bacteria have been determined previously (Mahizan et al., 2019). Therefore, the acyclic terpenes produced by both strains could contribute to their high antibacterial activity against both Gram-positive and Gram-negative bacteria. The cyclic terpenes or their derivatives were not detected in the VOC mixtures of both cave strains, despite the fact that the gene of terpene cyclase (Dickschat, 2016) was identified in the respective BGCs.

In addition, both of the cave strains were found to produce a semivolatile cyclic dipeptide cyclo(Pro-Phe). The antibacterial, antifungal, antitumor, and antiviral activities of the cyclic dipeptides have been well documented (de Carvalho and Abraham, 2012; Mishra et al., 2017). Therefore, this compound may contribute to the high antibacterial activity of both cave strains. NRPSs are known to be involved in the biosynthesis of cyclic dipeptides through the premature release of dipeptidyl intermediates (Goering et al., 2017; Mishra et al., 2017). Therefore, we compared the genome mining results with those of GC-MS analysis. However, the involvement of NRPS in the biosynthesis of cyclo(Pro-Phe) was rejected, since it did not possess the two ADs, one after another, specific to proline and phenylalanine.

## Conclusion

In summary, our study showed that the endospore-forming cave strains of the genus *Paenibacillus* have a high potential for the production of a wide range of bioactive compounds. Although some BGCs could be attributed to the biosynthesis of known compounds (fusaricidin, polymyxin B, colistin A, tridecaptin, and paenicidin B) of this genus, most BGCs have been predicted to represent novel biosynthetic pathways. In addition, the cave strains differed in their potential to encode the biosynthesis of a few unique, previously unknown compounds. These findings indicate that studies on distinct strains are not only reasonable but also valuable. Thus, more attention should be paid to the endospore-forming bacteria in future studies on the bioactivity of the cave microbiomes.

## Data Availability Statement

The datasets presented in this study can be found in online repositories. The names of the repository/repositories and accession number(s) can be found below: https://www.ncbi.nlm. nih.gov/genbank/, MK511842 https://www.ncbi.nlm.nih.gov/genbank/, MK511843 https://www.ncbi.nlm.nih.gov/genbank/, SPQH00000000.1 https://www.ncbi.nlm.nih.gov/genbank/, SLTL 00000000.1.

## Author Contributions

JL, VV, RP, and NK designed the experiments. JL, GJ, RČ, VV, and RP performed the research and analyzed the data. NK directed the research, analyzed the data, and wrote the manuscript. All authors contributed to the article and approved the submitted version.

## Conflict of Interest

The authors declare that the research was conducted in the absence of any commercial or financial relationships that could be construed as a potential conflict of interest.

## Footnotes

1 http://ggdc.dsmz.de/

2 http://bioinformatics.charite.de/mvoc/

3 https://antismash.secondarymetabolites.org/#!/start

4 http://bagel4.molgenrug.nl/links.php

5 https://blast.ncbi.nlm.nih.gov/Blast.cgi

6 https://mibig.secondarymetabolites.org/

7 http://bactibase.hammamilab.org/main.php

8 http://npdomainseeker.sdsc.edu//napdos_home.html

9 https://www.ncbi.nlm.nih.gov/tools/primer-blast/index.cgi?LINK_LOC=BlastHome

10 http://cels.uri.edu/gsc/cndna.html

11 http://www.heatmapper.ca/
