# Supplementary material for: Genome Mining and Characterization of Biosynthetic Gene Clusters in Two Cave Strains of Paenibacillus sp
Source: Front Microbiol. 2021 Jan 11;11:612483. doi: 10.3389/fmicb.2020.612483 (PMC7829367; doi:10.3389/fmicb.2020.612483)
Supplement: Supplementary file 1 [file Data_Sheet_1.PDF]

Supplementary Material

SUPPLEMENTARY TABLE S1. Results of GC-MS analysis of the volatile and semivolatile compounds of the strains 23TSA30-6 and 28ISP30-2.

PubChem CID	Compound	Strain	R match	mVOC
985	hexadecanoic acid	23TSA30-6<br>28ISP30-2	895<br>872	*Bacillus* sp.; *Bacteroides fragilis; Bacteroides gracilis; Bacteroides ureolyticus; Campylobacter fetus* subsp. *venerealis*; *Porphyromonas endodontalis; Prevotella buccae; Prevotella disiens; Prevotella heparinolyticus; Prevotella oralis; Prevotella oris; Prevotella veroralis; Pseudomonas simiae; Wolinella curva; Wolinella recta; Wolinella succinogenes;* marine streptomycete
5281	octadecanoic acid	23TSA30-6<br>28ISP30-2	921<br>817	*Bacteroides gracilis; Bacteroides ureolyticus; Campylobacter fetus* subsp. *venerealis*; *Pseudomonas simiae; Wolinella curva; Wolinella recta; Wolinella succinogenes;* marine streptomycete
6782	bis(2-methylpropyl) benzene-1,2-dicarboxylate	23TSA30-6<br>28ISP30-2	944<br>843	-
7311	2,4-ditert-butylphenol	23TSA30-6<br>28ISP30-2	928<br>910	*Arthrobacter agilis; Shewanella algae*

10441	nonadecan-10-one	23TSA30-6<br>28ISP30-2	862<br>836	*Stigmatella aurantiaca*
11006	hexadecane	23TSA30-6<br>28ISP30-2	921<br>880	*Arthrobacter agilis; Bacillus megaterium; Bacillus simplex; Bacillus subtilis; Bacillus weihenstephanensis; Microbacterium oxydans; Pseudomonas putida; Serratia marcescens; Stenotrophomonas maltophilia; Streptomyces lateritius*; cyanobacteria
11635	octadecane	23TSA30-6<br>28ISP30-2	883<br>864	*Azospirillum brasilense; Bacillus pumilus; Escherichia coli; Pseudomonas brassicacearum; Streptococcus mutans*
12389	tetradecane	23TSA30-6<br>28ISP30-2	934<br>936	*Bacillus simplex; Bacillus subtilis; Bacillus weihenstephanensis; Microbacterium oxydans; Pseudomonas aurantiaca; Pseudomonas chlororaphis; Pseudomonas corrugata; Pseudomonas fluorescens; Pseudomonas putida; Pseudomonas simiae; Serratia marcescens; Stenotrophomonas maltophilia; Streptomyces lateritius*
12395	hexadec-1-ene	23TSA30-6<br>28ISP30-2	938<br>914	*Carnobacterium divergens; Serratia proteamaculans; Pseudomonas fragi*
12398	heptadecane	23TSA30-6<br>28ISP30-2	848<br>867	*Bacillus megaterium; Calothrix* sp.; *Phormidium* sp.; *Plectonema* sp.; *Pseudomonas brassicacearum;*

				Pseudomonas putida; Pseudomonas simiae; Rivularia sp.; *Tolypothrix* sp.
18936	icos-1-ene	23TSA30-6	919	-
		28ISP30-2	858	
31292	octadecanamide	23TSA30-6	831	*Pseudomonas simiae*
		28ISP30-2	819	
35768	2,6,11-trimethyldodecane	23TSA30-6	830	*Klebsiella pneumoniae; Pseudomonas brassicacearum; Pseudomonas putida*
		28ISP30-2	846	
93447	2,7,10-trimethyldodecane	23TSA30-6	832	-
		28ISP30-2	843	
99895	3-benzyl-2,3,6,7,8,8a-hexahydropyrrolo[1,2-a] pyrazine-1,4-dione	23TSA30-6	903	-
		28ISP30-2	894	
136331	2,6,11,15-tetramethylhexadecane	23TSA30-6	844	-
		28ISP30-2	813	
136810	1,3-ditert-butylbenzene	23TSA30-6	906	-
		28ISP30-2	911	
229159	undecyl benzoate	23TSA30-6	818	-

		28ISP30-2	845	
530070	10-methylnonadecane	23TSA30-6	891	*Pseudomonas putida*
		28ISP30-2	895	
545303	7,9-ditert-butyl-1-oxaspiro[4.5] deca-6,9-diene-2,8-dione	23TSA30-6	940	*Pseudomonas putida*
		28ISP30-2	860	
5283387	(*Z*)-octadec-9-enamide	23TSA30-6	896	-
		28ISP30-2	852	
3026	dibutyl benzene-1,2-dicarboxylate	23TSA30-6	854	*Carnobacterium divergens; Pseudomonas fragi*
8217	octadec-1-ene	23TSA30-6	859	-
13162	pentadecan-8-one	23TSA30-6	906	-
69421	hexadecanamide	23TSA30-6	856	-
5365075	(*Z*)-tricos-9-ene	23TSA30-6	845	-
19775	2,6,10-trimethylpentadecane	28ISP30-2	813	-
28454	4,8-dimethylundecane	28ISP30-2	852	-
29036	2,6,10,14-tetramethylheptadecane	28ISP30-2	828	-

41209	2,6,10,15-tetramethylheptadecane	28ISP30-2	808	-
520323	5-ethyl-5-propylundecane	28ISP30-2	810	-
545627	4,6-dimethyldodecane	28ISP30-2	830	-
609923	(1-methyl-2,2-diphenylcyclopropyl) sulfanylbenzene	28ISP30-2	821	-
